# Effectiveness of diffusion tensor imaging in differentiating early-stage subcortical ischemic vascular disease, Alzheimer’s disease and normal ageing

**DOI:** 10.1371/journal.pone.0175143

**Published:** 2017-04-07

**Authors:** Min-Chien Tu, Chung-Ping Lo, Ching-Feng Huang, Yen-Hsuan Hsu, Wen-Hui Huang, Jie Fu Deng, Yung-Chuan Lee

**Affiliations:** 1 Department of Neurology, Taichung Tzu Chi Hospital, Buddhist Tzu Chi Medical Foundation, Taichung, Taiwan; 2 Department of Radiology, Taichung Tzu Chi Hospital, Buddhist Tzu Chi Medical Foundation, Taichung, Taiwan; 3 Department of Psychology, National Chung Cheng University, Chiayi, Taiwan; 4 Department of Business Administration, Asia University, Taichung, Taiwan; University of California, San Francisco, UNITED STATES

## Abstract

**Objective:**

To describe and compare diffusion tensor imaging (DTI) parameters between patients with subcortical ischemic vascular disease (SIVD) and Alzheimer’s disease (AD) diagnosed using structuralized neuropsychiatric assessments, and investigate potential neuronal substrates related to cognitive performance.

**Methods:**

Thirty-five patients with SIVD, 40 patients with AD, and 33 cognitively normal control (NC) subjects matched by age and education level were consecutively recruited and underwent cognitive function assessments and DTI examinations. Comparisons among these three subgroups with regards to cognitive performance and DTI parameters including fractional anisotropy (FA) and mean diffusivity (MD) values were performed. Partial correlation analysis after controlling for age and education was used to evaluate associations between cognitive performance and DTI parameters.

**Results:**

With regards to cognitive performance, the patients with SIVD had lower total scores in frontal assessment battery (FAB) compared to those with AD (*p* < 0.05) in the context of comparable Mini-Mental Status Examination and Cognitive Abilities Screening Instrument scores. With regards to DTI parameters, there were more regions of significant differences in FA among these three subgroups compared with MD. Compared with NC group, the patients with SIVD had significant global reductions in FA (*p* < 0.001 ~ 0.05), while significant reductions in FA among the patients with AD were regionally confined within the left superior longitudinal fasciculus, genu and splenium of the corpus callosum, and bilateral forceps major, and the anterior thalamic radiation, uncinate fasciculus, and cingulum of the left side (*p* < 0.01 ~ 0.05). Analysis of FA values within the left forceps major, left anterior thalamic radiation, and genu of the corpus callosum revealed a 71.8% overall correct classification (*p* < 0.001) with sensitivity of 69.4%, specificity of 73.8%, positive predictive value of 69.4%, and negative predictive value of 73.8% in discriminating patients with SIVD from those with AD. In combined analysis of the patients with SIVD and AD (*n* = 75), the total FAB score was positively correlated with FA within the bilateral forceps minor, genu of the corpus callosum, left forceps major, left uncinate fasciculus, and right inferior longitudinal fasciculus (*p* = 0.001 ~ 0.038), and inversely correlated with MD within the right superior longitudinal fasciculus, genu and body of the corpus callosum, bilateral forceps minor, right uncinate fasciculus, and right inferior longitudinal fasciculus (*p* = 0.003 ~ 0.040)

**Conclusions:**

Our findings suggest the effectiveness of DTI measurements in distinguishing patients with early-stage AD from those with SIVD, with discernible changes in spatial distribution and magnitude of significance of the DTI parameters. Strategic FA assessments provided the most robust discriminative power to differentiate SIVD from AD, and FAB may serve as an additional cognitive marker. We also identified the neuronal substrates responsible for FAB performance.

## Introduction

Dementia is a syndrome in which there is deterioration in memory, thinking, behavior and the ability to perform everyday activities [1]. Diagnosing the subtype of dementia generally requires a clinical profile, neuropsychological assessments, and neuroimaging studies. Although Alzheimer’s disease (AD) and vascular cognitive impairment (VCI) constitute the majority of patients with dementia [2], diagnosing and differentiating the subtypes of dementia, particularly during the early stage, remains a challenge. For example, both AD and VCI may present with an insidious onset and progressive course. When a history of prior clinical stroke is unavailable, the differential diagnosis appears to be somewhat difficult. In addition to the slow rate of disease progression, there is also considerable overlap between AD and VCI with regards to their underlying pathology, neuropsychological profile, and neuroimaging features. In pathological reports, ischemic changes and arteriosclerosis have been reported in patients with AD [3,4], and about one third of patients diagnosed with VCI may have AD-type pathology at autopsy [5]. In neuropsychological tests, although some researchers have concluded that patients with AD and VCI have their own specific characteristic cognitive profiles [6–8], others have reported a poor diagnostic accuracy in differentiating dementia subtypes [9,10]. This inconsistency may partly be because VCI encompasses a group of heterogeneous pathological properties (e.g., ischemia, micro/macro-hemorrhage) and involves different regions (e.g., cortex, subcortical regions, and strategic infarcts) with variable severity (e.g., local, multifocal, and diffuse lesions) [11]. The diagnosis of subcortical ischemic vascular dementia (SIVD) appears to be associated with a pathology more confined within subcortical regions, and with lacunes and white matter changes involving cognitive impairment. Therefore the pathogenesis of SIVD appears to be more homogeneous. Typical neuroimaging features of patients with SIVD include hyperintensities extending into periventricular and deep white matter on T2-weighted magnetic resonance imaging (MRI), and lacunes within the deep gray matter [12]. However, both periventricular and deep white matter hyperintensities have also been documented in patients with AD [13,14] and in normal elderly subjects [15,16], making it a challenge to differentiate between SIVD and AD in the early stage, and even more for normal ageing process.

Diffusion tensor imaging (DTI) has shown promise in evaluating white matter integrity and microstructural changes within regions that commonly have a normal appearance in conventional MRI [17]. In the ageing and/or pathological process, the diffusion of water within the affected brain tissues is thought to be altered by changes in the tissue microstructure and organization. DTI parameters such as fractional anisotropy (FA) and mean diffusivity (MD) are potentially powerful probes to characterize changes both at the cellular and microstructural level. While an increasing amount of research has focused on evaluating and comparing pathological processes in patients with AD and mild cognitive impairment [18–20], comparisons between patients with VCI and AD are relatively rare [21,22], and comparisons between SIVD and AD even more so [23].

The aims of the current study were to (i) describe and compare the DTI parameters between SIVD and AD in the context of structuralized neuropsychiatric assessments, and (ii) identify potential regions related to cognitive performance using DTI parameters.

## Materials and methods

Thirty-five patients with SIVD and 40 patients with AD who visited the Department of Neurology of our hospital from July 2014 to June 2016 were consecutively recruited. Data on demographics, serology tests, general cognitive function assessments, and brain MRI (including DTI) studies were recorded for each patient.

Another 33 cognitively-normal subjects matched by age and education level were recruited as the normal control (NC) group for comparisons of cognitive function and DTI parameters. The study was approved by the Institutional Review Board of our hospital (REC 103–14). All participants provided their written informed consent to participate in this study.

### Inclusion and exclusion criteria

The inclusion criteria for the patients with SIVD were: (1) cognitive complaints with interference in complex occupational and social activities [12]; (2) evidence of subcortical ischemic changes in brain MRI [12]; (3) Clinical Dementia Rating (CDR) = 0.5 ~ 1 [24]; (4) Mini-Mental State Examination (MMSE) score ≤ 26 [25]; and (5) Hachinski Ischemic Scale score ≥ 7 [26]. The inclusion criteria for the patients with AD were: (1) changes in cognition reported by the patient, informant or clinician [27]; (2) absence of profound subcortical ischemic change in brain MRI [12]; (3) CDR = 0.5 ~ 1 [24]; (4) MMSE score ≤ 26 [25]; and (5) Hachinski Ischemic Scale score ≤ 4 [26].

The exclusion criteria were: (1) state of delirium; (2) stroke event within 2 weeks; (3) appearance of cortical and/or cortico-subcortical non-lacunar territorial infarcts and watershed infarcts, hemorrhages, signs of normal pressure hydrocephalus, and specific causes of white matter lesions (e.g. multiple sclerosis, sarcoidosis, brain irradiation) [12]; (4) derangements in serology tests contributing to cognitive impairment (e.g. abnormal levels of free T4, cortisol, folic acid, vitamin B12, or rapid plasma reagin); and (5) severe hearing or visual impairment.

### Demographic data registry

Disease duration, defined by time interval between initial cognitive symptom (e.g., frequent missing an appointment, forgetfulness, poor efficiency on work, etc.) and the first clinic visit, was recorded. The systemic diseases of all patients were registered. Hypertension was defined as a systolic blood pressure ≥ 140 mmHg or a diastolic blood pressure ≥ 90 mmHg at two separate blood pressure measurements [28], self-report of a diagnosis of hypertension, or medical treatment for hypertension. Diabetes mellitus was defined as a fasting blood sugar level ≥ 126 mg/dl, random postprandial blood sugar level ≥ 200 mg/dl, HbA1C ≥ 6.5% [29], self-report of a diagnosis of diabetes mellitus, or treatment with insulin or oral hypoglycemic agents. Chronic kidney disease was defined as a glomerular filtration rate according to the Modification of Diet in Renal Disease Study equation [30] < 60 mL per minute per 1.73 m2 for ≥ 3 months with or without evidence of kidney damage [31]. Coronary artery disease was defined as events and/or history related to stable angina pectoris, unstable angina pectoris, or a myocardial infarction [32]. To avoid the confounding effect of medications on cognitive performance and neuropsychiatric symptoms, the current use (within 1 month) of antipsychotics, anxiolytics, and antidepressants was reviewed and recorded.

### Serology tests

Antecubital venous blood samples were collected after an 8-hour fast for hemogram, serum creatinine, folate, vitamin B12, free T4, thyroid stimulating hormones, cortisol, and rapid plasma reagin measurements. Samples were collected in evacuated tubes containing EDTA, centrifuged within 10 minutes and stored below -20°C until analysis.

### Cognitive function assessment

General cognitive evaluations included CDR [24], the Taiwanese version of the MMSE [25], and Cognitive Abilities Screening Instrument (CASI) [14]. The CDR is a semi-structured interview with the patient and a reliable informant. It characterizes six domains of cognitive and functional performance including memory, orientation, judgment and problem solving, community affairs, home and hobbies, and personal care. An overall score is reached according to a standardized algorithm. A CDR score of 0 denotes no cognitive impairment, with the remaining four scores representing various stages of severity (0.5: very mild; 1: mild; 2: moderate; 3: severe) [24]. Both the MMSE and CASI assess global cognition of the subjects, with a higher score representing better cognition.

Both the patients and NC subjects also received tests to evaluate individual cognitive domains. For the memory domain, the Chinese Version Verbal Learning Test (CVVLT) (i.e., immediate recall, 30-second delay, 10-minute delay, cued recall, and recognition as index scores) [33] and Wechsler Memory Scale (WMS) (i.e., recall I, recall II, and recognition as index scores) [34] were assessed. For the attention domain, the total score and forward subset of digit span [34] were evaluated. For the execution domain, the backward subset of digit span [34] and the total score of Frontal Assessment Battery (FAB) [35] were evaluated. For the visuospatial construct domain, the total score of the clock drawing tests [36] was recorded. For the language domain, the subset of category fluency and language score from the CASI [14] were recorded. A higher score in each individual test represented better cognitive performance.

### Brain MRI

All patient received brain MRI using a 3.0 T scanner (Discovery MR750, GE Medical System, Milwaukee, WI) with an 8-channel phased array head coil in accordance with same imaging protocol, including axial T1-weighted imaging, T2 fluid-attenuated inversion recovery [T2-FLAIR], diffusion weighted imaging, and MR angiography for the circle of Willis. White matter hyperintensities were rated according to the Fazekas scale [37] from T2-FLAIR sequences. The Fazekas scale classifies white matter into periventricular and deep white matter, and each is given a grade (0 to 3) depending on the size and confluence of quantification of the lesions [37]. DTI data were acquired using a single-shot spin-echo echo-planar imaging sequence. The diffusion-sensitizing gradients were applied along 20 non-collinear directions with diffusion weighting factor b = 1000 sec/mm^2^, plus one b = 0 image. The imaging parameters were: TR/TE = 8000/82 msec, matrix size = 128x128, field of view (FOV) = 240 mm, slice thickness = 3 mm with no intersection gap, number of excitations = 2, number of slices = 67, scan time = 5 minutes and 58 seconds.

**Fig 1:**
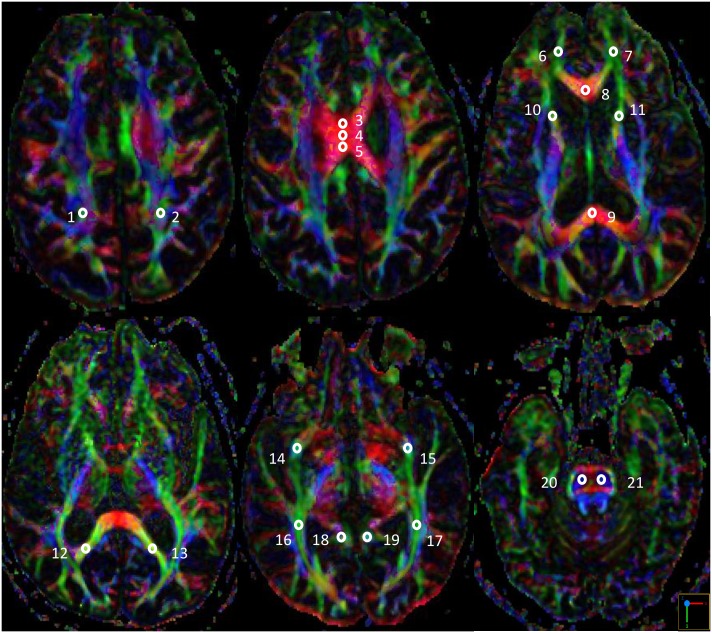
Illustrations of diffusion tensor imaging template. 1/2: Right/Left superior longitudinal fasciculus; 3/4/5: body (three portions) of the corpus callosum; 6/7: Right/Left forceps minor; 8/9: the genu/splenium of the corpus callosum; 10/11: Right/Left anterior thalamic radiation; 12/13: Right/Left forceps major; 14/15: Right/Left uncinate fasciculus; 16/17: Right/Left inferior longitudinal fasciculus; 18/19: Right/Left cingulum; 20/21: Right/Left corticospinal tract at mid-pons level.

The post-processing software, Functool, was used to measure FA and MD values in different slices of B0 and color-coded maps on the axial images in the three groups. FA and MD were measured from 21 circular priori-defined subcortical regions of interest (ROI). FA values ranged from zero to one, with a higher value indicating a greater degree of white matter integrity. In contrast, a higher MD value indicated a greater degree of white matter damage. The ROI size was kept consistent (30–35 mm^2^) in all of the patients in order to obtain a stable number of voxels and decrease variance in the DTI parameters. The positions of the ROIs were compared to the corresponding slices of the T2-FLAIR axial data sets to avoid measurements within regions of lacunes or hyperintensities. Midline ROIs included the genu, body (three portions) and splenium of the corpus callosum. Other ROIs including the superior longitudinal fasciculus, forceps minor, forceps major, anterior thalamic radiation, uncinate fasciculus, inferior longitudinal fasciculus, and cingulum, were evaluated symmetrically within bilateral hemispheres (the template is shown in Fig 1). Additionally, bilateral corticospinal tracts were also assessed at the mid-pons level as the control. The ROIs were manually drawn by a single rater (Min-Chien Tu). The DTI parameters of each white matter tract were obtained from the averaged ROIs of two adjacent slices.

### Statistical analysis

Analysis of variance (ANOVA) and the *χ*^2^ test were used to detect group differences in demographic data, cognitive function assessments and DTI parameters where appropriate. Spearman rank correlation test was used to examine the relationship between Fazekas scale and DTI parameters. Multivariate analysis of covariance (MANCOVA) was used to compare differences of DTI parameters across a covariate. Partial correlation analysis after controlling for age and education was used to evaluate associations between the cognitive test scores and DTI parameters. To identify discriminating variables between SIVD and AD, a stepwise discriminant analysis was performed. In this analysis, dementia subgroup (SIVD or AD) was the dependent variable and the potential DTI parameters and cognitive performances as well as demographic data were the independent variables. All statistical tests were performed using SPSS software version 19 (IBM, Armonk, New York). A *p* value less than 0.05 was considered to be statistically significant.

## Results

### Demographic data

Table 1 shows the demographic data of the AD, SIVD, and NC groups. Both patients with AD and SIVD had disease duration ranging from 0.5 to 2 years. In the SIVD group, 22 patients had a CDR score of 0.5 and 13 had a CDR score of 1, compared to 26 and 14, respectively, in the patients with AD. No significant difference was found with regards to the CDR severity score (*X*^2^ = 0.0372, *p =* 0.847) and disease duration (*p* = 0.423). The patients with SIVD had higher Hachinski and Fazekas scale scores than those with AD or the NC (*p* < 0.001). Similarly, more patients with SIVD had a history of cerebrovascular diseases than those with AD (*p* < 0.01). In addition, more of the SIVD group were male compared to the other two groups (*p* < 0.05). In a review of current medications, more of the patients with AD used anxiolytics than those with SIVD (*p* < 0.01).

**Table 1:** Demographic data of the study participants.

	SIVD	AD	NC	ANOVA	
	(*n* = 35)	(*n* = 40)	(*n* = 33)	*F*	*p*
Age	73.3 (11.3)	72.2 (8.6)	69.9 (12.2)	0.905	0.408
Education	6.8 (4.2)	5.7 (4.0)	7.3 (4.6)	1.430	0.244
Disease duration (year)	1.2 (0.7)	1.0 (0.6)	0.0 (0.0)	48.279	< 0.001^A^^B^
Hachinski score	7.1 (3.0)	1.1 (0.9)	0.6 (0.7)	136.952	<0.001 ^A^^C^
Fazekas scale (Total)	4.7 (1.0)	2.1 (1.2)	1.8 (1.1)	73.535	<0.001 ^A^^C^
Periventricular white matter	2.3 (0.6)	1.1 (0.6)	1.0 (0.6)	52.423	< 0.001 ^A^^C^
Deep white matter	2.3 (0.6)	1.0 (0.9)	0.8 (0.8)	42.516	< 0.001 ^A^^C^
				Chi-Square tests	
				*X*^2^	*p*
Gender (M/F)	27/8	20/20	16/17	5.878–6.000	0.015–0.014^a^^c^
Systemic diseases (*n*)					
Cerebrovascular disease	12	3	-	8.371	0.004^*C*^
Hypertension	17	12	-	2.715	0.099
Diabetes mellitus	12	7	-	2.781	0.095
Chronic kidney disease	7	7	-	0.077	0.782
Medications (*n*)					
Antipsychotics	0	1	-	0.887	0.346
Anxiolytics	0	7	-	6.756	0.009^*C*^
Antidepressants	4	5	-	0.020	0.887

^A^:*p*<0.001 on comparison between SIVD and the control;

^a^:*p*<0.05 on comparison between SIVD and the control;

^B^:*p* <0.001 on comparison between AD and the control;

^C^:*p*<0.001 on comparison between SIVD and AD;

^***C***^:*p*<0.01 on comparison between SIVD and AD;

^c^:*p*<0.05 on comparison between SIVD and AD.

Data presented as Mean (Standard deviation) unless stated elsewhere. AD: Alzheimer’s disease; SIVD: Subcortical ischemic vascular disease; NC: Normal Control; ANOVA: Analysis of variance.

### Comparisons of cognitive assessments

Table 2 shows the results of cognitive assessments among the three groups. Compared to the NC, the patients with SIVD and AD had lower MMSE and CASI scores and higher CDR-sum of box scores (*p* < 0.01 ~ 0.001). Cognitive tests of individual domains showed significantly worse performances among the patients with SIVD compared to the NC (*p* < 0.05 ~ 0.001) except for recall II subset of the WMS and clock drawing test. In comparison, the patients with AD had significantly lower scores in the CVVLT (*p* < 0.05 ~ 0.001), WMS-recognition (*p* < 0.01), total score and forward performance of digit span (both *p* < 0.01), FAB total score (*p* < 0.05), and language score (*p* < 0.01) than the NC. Of note, the patients with SIVD had lower total FAB scores compared to those with AD (*p* < 0.05).

**Table 2:** Cognitive assessment of the study participants.

	SIVD	AD	NC	ANOVA	
	(*n* = 35)	(*n* = 40)	(*n* = 33)	*F*	*p*
Mini-Mental State Examination	20.6 (5.6)	21.8 (4.7)	25.8 (2.9)	11.475	<0.001^A^^*B*^
Cognitive Abilities Screening Instrument	64.8 (17.6)	69.3 (15.8)	81.9 (10.2)	11.918	<0.001^A^^*B*^
Clinical Dementia Rating – sum of box	3.7 (3.2)	3.4 (2.4)	0.0 (0.0)	19.178	<0.001^A^^B^
**Memory** CVVLT – immediate recall	16.5 (6.0)	18.1 (4.1)	21.1 (6.1)	6.316	0.003^*A*^^b^
– 30 seconds delay	4.0 (2.1)	4.1 (2.4)	6.5 (1.4)	16.396	<0.001^A^^B^
– 10 minutes delay	3.0 (2.9)	2.7 (2.8)	5.4 (2.3)	10.867	<0.001^*A*^^B^
– cued recall	4.5 (2.5)	3.8 (2.5)	6.5 (1.9)	13.063	<0.001^*A*^^B^
– recognition	6.6 (2.9)	6.7 (2.8)	8.3 (1.1)	5.267	0.007^a^^b^
WMS – recall I	41.4 (29.5)	44.1 (21.6)	58.0 (24.9)	3.856	0.025^a^
– recall II	14.1 (19.9)	15.5 (23.2)	24.9 (21.5)	2.051	0.135
– recognition	33.5 (8.6)	34.2 (6.0)	39.6 (4.0)	8.419	<0.001^*A*^^*B*^
**Attention** Digit span – total score	12.6 (4.3)	13.7 (4.3)	16.9 (4.2)	8.540	<0.001^A^^*B*^
– forward	9.2 (3.0)	9.6 (3.0)	11.8 (2.3)	8.009	0.001^*A*^^*B*^
**Execution** Digit span – backward	3.6 (2.2)	4.1 (2.1)	5.3 (2.5)	4.480	0.014^a^
FAB total	8.7 (3.0)	10.7 (3.2)	12.6 (3.2)	11.999	<0.001^A^^b^^c^
**Visuospatial construct** Clock drawing	2.3 (1.4)	2.2 (1.3)	1.8 (1.0)	1.154	0.320
**Language** Language score	7.9 (1.6)	8.6 (1.6)	9.9 (2.0)	11.121	<0.001^A^^*B*^
Verbal fluency	4.3 (2.8)	5.5 (2.4)	6.6 (2.1)	7.607	0.002^A^

^A^:*p* <0.001 on comparison between SIVD and the control;

^***A***^:*p* <0.01 on comparison between SIVD and the control;

^a^:*p* <0.05 on comparison between SIVD and the control;

^B^:*p* <0.001 on comparison between AD and the control;

^***B***^:*p* <0.01 on comparison between AD and the control;

^b^:*p* <0.05 on comparison between AD and the control;

^c^:*p* <0.05 on comparison between SIVD and AD.

Data presented as *Mean* (*Standard deviation*). AD: Alzheimer’s disease; SIVD: Subcortical ischemic vascular disease; NC: Normal Control; ANOVA: Analysis of variance; CVVLT: Chinese Version Verbal Learning Test; WMS: Wechsler Memory Scale; FAB: Frontal Assessment Battery.

### Comparisons of DTI parameters

Table 3 shows comparisons of FA among the three groups. Except for the left forceps minor and right uncinate fasciculus, patients with SIVD had significantly lower FA values than the NC (*p* < 0.05 ~ 0.001). In comparison, the patients with AD had fewer ROIs where the FA was lower than the NC, including the left superior longitudinal fasciculus (*p* < 0.05), genu and splenium of the corpus callosum (both *p* < 0.05), bilateral forceps major (both *p* < 0.01), and the anterior thalamic radiation, uncinate fasciculus, and cingulum of the left side (all *p* <0.05).

**Table 3:** Fractional anisotropy of the study participants.

	SIVD	AD	NC	ANOVA	
Regions of Interest	(*n* = 35)	(*n* = 40)	(*n* = 33)	*F*	*p*
Superior longitudinal fasciculus – Rt	0.38 (0.07)	0.46 (0.09)	0.46 (0.09)	11.307	<0.001^A^^C^
– Lt &	0.39 (0.09)	0.44 (0.09)	0.50 (0.11)	9.991	<0.001^A^^b^
Corpus callosum – Genu $$&	0.59 (0.14)	0.69 (0.12)	0.76 (0.07)	19.040	<0.001^A^^b^^C^
– Body 1	0.56 (0.15)	0.65 (0.12)	0.65 (0.12)	5.369	0.006^a^^c^
– Body 2	0.54 (0.18)	0.62 (0.13)	0.64 (0.13)	4.484	0.014^a^
– Body 3	0.57 (0.16)	0.64 (0.13)	0.67 (0.11)	4.980	0.009^a^^c^
– Splenium $&	0.57 (0.16)	0.66 (0.14)	0.74 (0.10)	12.978	<0.001^A^^b^^c^
Forceps minor – Rt	0.34 (0.05)	0.40 (0.07)	0.40 (0.07)	10.347	<0.001 ^A^^*C*^
– Lt	0.34 (0.07)	0.37 (0.06)	0.37 (0.07)	2.164	0.120
Forceps major – Rt $&&	0.37 (0.09)	0.41 (0.08)	0.48 (0.08)	16.162	<0.001^A^^*B*^
– Lt &&	0.36 (0.08)	0.43 (0.08)	0.50 (0.10)	23.223	<0.001^A^^*B*^^*C*^
Anterior thalamic radiation – Rt	0.34 (0.09)	0.40 (0.09)	0.43 (0.08)	8.624	<0.001^A^^c^
– Lt $&	0.32 (0.12)	0.39 (0.09)	0.46 (0.08)	16.785	<0.001^A^^b^^*C*^
Uncinate fasciculus – Rt	0.30 (0.09)	0.35 (0.09)	0.35 (0.10)	3.174	0.046
– Lt $$&	0.30 (0.08)	0.35 (0.10)	0.41 (0.09)	11.224	<0.001^A^^b^
Inferior longitudinal fasciculus – Rt	0.43 (0.09)	0.49 (0.07)	0.52 (0.07)	10.925	<0.001^A^^*C*^
– Lt $$&††	0.47 (0.10)	0.55 (0.07)	0.58 (0.06)	16.597	<0.001^A^^C^
Cingulum – Rt $$$††	0.38 (0.07)	0.44 (0.06)	0.47 (0.06)	16.839	<0.001^A^^*C*^
– Lt $$&	0.38 (0.06)	0.40 (0.07)	0.45 (0.07)	6.741	0.002 ^*A*^^b^
Corticospinal tract – Rt	0.45 (0.06)	0.48 (0.06)	0.50 (0.08)	3.065	0.053
– Lt	0.46 (0.06)	0.44 (0.06)	0.46 (0.05)	0.892	0.414

^A^:*p* <0.001 on comparison between SIVD and the control;

^***A***^:*p* <0.01 on comparison between SIVD and the control;

^a^:*p* <0.05 on comparison between SIVD and the control;

^B^:*p* <0.001 on comparison between AD and the control;

^***B***^**:***p* <0.01 on comparison between AD and the control;

^b^:*p* <0.05 on comparison between AD and the control;

^C^:*p* <0.001 on comparison between SIVD and AD;

^***C***^:*p* <0.01 on comparison between SIVD and AD.

^c^:*p* <0.05 on comparison between SIVD and AD.

Through MANCOVA with Fazekas scale as a covariate,

^$$$^:*p* <0.001 on comparison between SIVD and the control;

^$$^:*p* <0.01 on comparison between SIVD and the control;

^$^:*p* <0.05 on comparison between SIVD and the control;

^&&^**:***p* <0.01 on comparison between AD and the control;

^&^:*p* <0.05 on comparison between AD and the control;

^††^:*p* <0.01 on comparison between SIVD and AD.

Data presented as *Mean* (*Standard deviation*). AD: Alzheimer’s disease; SIVD: Subcortical ischemic vascular disease; NC: Normal Control; ANOVA: Analysis of variance; MANCOVA: Multivariate analysis of covariance; Rt: Right; Lt: Left.

In comparisons between the patients with SIVD and AD, those with SIVD had lower FA values in the right superior longitudinal fasciculus (*p* < 0.001), genu of the corpus callosum (*p* < 0.001), the body (portion 1 and 3) and splenium of the corpus callosum (all *p* < 0.05), right forceps minor (*p* < 0.01), left forceps major (*p* < 0.01), bilateral anterior thalamic radiation (*p* < 0.05 ~ 0.01), bilateral inferior longitudinal fasciculus (*p* < 0.01 ~ 0.001), and right cingulum (*p* < 0.01). There was no significant difference within corticospinal tracts among the three groups. Among patient groups (SIVD + AD; *n* = 75), there were significant inverse correlations between Fazekas scale and FA values within multiple ROIs (*p* < 0.05 ~ 0.001) (S1 Appendix). On taking Fazekas scale as a covariate into account, the original results were modified, mostly in the pairwise comparisons between SIVD and AD.

Table 4 shows comparisons of MD among the three groups. Similar to FA, the patients with SIVD had higher MD values than the NC (*p* < 0.05 ~ 0.001). However, the patients with AD only showed a trend of higher MD values than the NC within most ROIs, and no significant differences were noted between the AD and NC groups. Comparisons between the patients with SIVD and AD also revealed that the SIVD group had higher MD values overall (*p* < 0.05 ~ 0.001) except for the body (portion 2 and 3) of the corpus callosum and left cingulum. There was no significant difference within corticospinal tracts among the three groups. Among patient groups (SIVD + AD; *n* = 75), there were significant positive correlations between Fazekas scale and MD values within multiple ROIs (*p* < 0.05 ~ 0.001) (S1 Appendix). In MANCOVA, the original results remarkably altered on taking Fazekas scale as a covariate into account.

**Table 4:** Mean diffusivity of the study participants.

Regions of Interest	SIVD	AD	NC	ANOVA	
	(*n* = 35)	(*n* = 40)	(*n* = 33)	*F*	*p*
Superior longitudinal fasciculus – Rt †	0.973 (0.151)	0.788 (0.063)	0.784 (0.097)	35.260	<0.001^A^^C^
– Lt	0.860 (0.210)	0.754 (0.067)	0.745 (0.077)	8.265	<0.001^*A*^^*C*^
Corpus callosum – Genu	1.171 (0.310)	0.976 (0.258)	0.933 (0.161)	8.833	<0.001^*A*^^*C*^
– Body 1	1.138 (0.264)	0.960 (0.217)	0.949 (0.252)	6.709	0.002^*A*^^*C*^
– Body 2	1.154 (0.320)	1.017 (0.267)	0.914 (0.204)	6.788	0.002^*A*^
– Body 3	1.123 (0.293)	0.998 (0.245)	0.914 (0.167)	6.460	0.002^*A*^
– Splenium	1.209 (0.309)	1.027 (0.291)	0.949 (0.169)	8.670	<0.001^A^^c^
Forceps minor – Rt	0.922 (0.072)	0.868 (0.084)	0.873 (0.098)	4.491	0.013^a^^c^
– Lt †	0.943 (0.100)	0.844 (0.057)	0.851 (0.068)	18.739	<0.001^A^^C^
Forceps major – Rt	0.902 (0.127)	0.821 (0.099)	0.791 (0.083)	10.452	<0.001^A^^*C*^
– Lt	0.896 (0.170)	0.788 (0.071)	0.789 (0.055)	11.271	<0.001^A^^C^
Anterior thalamic radiation – Rt	0.991 (0.171)	0.865 (0.114)	0.813 (0.080)	17.715	<0.001^A^^C^
– Lt	1.129 (0.489)	0.825 (0.172)	0.798 (0.095)	13.086	<0.001^A^^C^
Uncinate fasciculus – Rt &	1.040 (0.112)	0.960 (0.113)	0.902 (0.094)	14.287	<0.001^A^^*C*^
– Lt	0.972 (0.123)	0.895 (0.115)	0.858 (0.084)	9.585	<0.001^A^^*C*^
Inferior longitudinal fasciculus – Rt	1.240 (0.292)	1.085 (0.214)	1.009 (0.188)	8.612	<0.001^A^^c^
– Lt	1.038 (0.248)	0.894 (0.109)	0.844 (0.086)	13.208	<0.001^A^^*C*^
Cingulum – Rt $	0.986 (0.211)	0.868 (0.135)	0.814 (0.062)	11.574	<0.001^A^^*C*^
– Lt	0.875 (0.132)	0.828 (0.111)	0.805 (0.076)	3.470	0.035^a^
Corticospinal tract – Rt	0.777 (0.060)	0.739 (0.148)	0.720 (0.058)	1.621	0.205
– Lt	0.769 (0.073)	0.770 (0.055)	0.745 (0.052)	1.258	0.290

^A^:*p* <0.001 on comparison between SIVD and the control;

^*A*^:*p* <0.01 on comparison between SIVD and the control;

^a^:*p* <0.05 on comparison between SIVD and the control;

^B^:*p* <0.001 on comparison between AD and the control;

^*B*^:*p* <0.01 on comparison between AD and the control;

^b^:*p* <0.05 on comparison between AD and the control;

^C^:*p* <0.001 on comparison between SIVD and AD;

^*C*^:*p* <0.01 on comparison between SIVD and AD.

^c^:*p* <0.05 on comparison between SIVD and AD.

Through MANCOVA with Fazekas scale as a covariate,

^$^:*p* <0.05 on comparison between SIVD and the control;

^&^:*p* <0.05 on comparison between AD and the control;

^†^:*p* <0.05 on comparison between SIVD and AD.

Data presented as *Mean* (*Standard deviation*) expressed in units of m^2^s^−1^×10^−9^. AD: Alzheimer’s disease; SIVD: Subcortical ischemic vascular disease; NC: Normal Control; ANOVA: Analysis of variance; MANCOVA: Multivariate analysis of covariance; Rt: Right; Lt: Left.

### Discriminative power of DTI parameters between the SIVD and AD groups

In significant pairwise comparisons (SIVD *vs*. NC; AD *vs*. NC; SIVD *vs*. AD), only FA values within four ROIs were significantly different, including the genu of the corpus callosum, splenium of the corpus callosum, left forceps major, and left anterior thalamic radiation. In addition, FAB was the only significant cognitive assessment able to differentiate subgroups. We therefore analyzed the discriminative power (SIVD *vs*. AD) with regard to FA of these four ROIs, FAB, age, sex, and education in a stepwise procedure.

**Fig 2:**
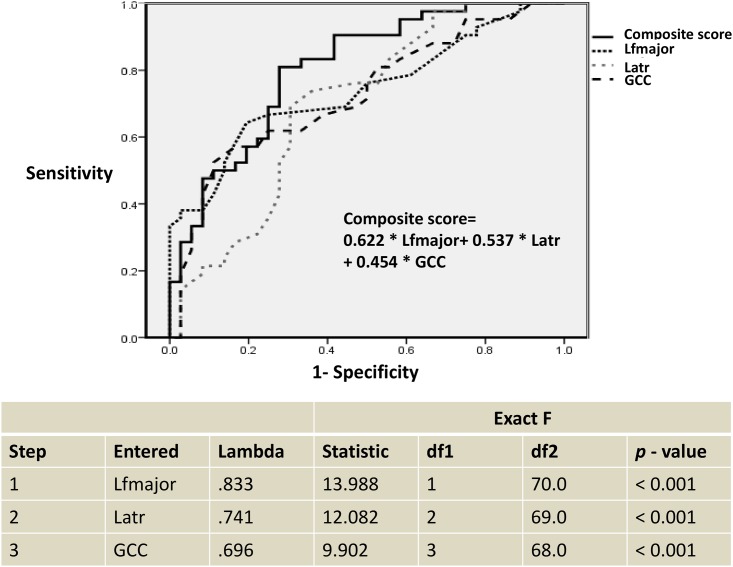
Stepwise discriminant analysis and receiver operating characteristic curve of dementia subgroup differentiation. Lfmajor: the left forceps major; Latr: the left anterior thalamic radiation; GCC: the genu of the corpus callosum.

The results showed that the combination of FA values of three ROIs, including the left forceps major, left anterior thalamic radiation, and genu of the corpus callosum best discriminated between SIVD and AD (Wilks’ lambda = 0.696, *p* < 0.001) (Fig 2). FAB, age, sex, and education didn’t enter the model. The model demonstrated that the left forceps major best discriminated between the dementia subgroups with a standardized discriminant function coefficient of 0.622, followed by a coefficient of 0.537 for the left anterior thalamic radiation and a coefficient of 0.454 for the genu of the corpus callosum. The average squared canonical correlation was 0.742, showing that these three variables, accounted for 74.2% of the overall variance in the data set.

The discriminative function equation (composite score) was as follows: 0.622 * the left forceps major + 0.537 * the left anterior thalamic radiation + 0.454 * the genu of the corpus callosum. Receiver operating characteristic curve was shown in Fig 2.

The results revealed a 71.8% overall correct classification (*p* < 0.001) in discriminating patients with SIVD from those with AD, with a sensitivity of 69.4%, specificity of 73.8%, positive predictive value of 69.4%, and negative predictive value of 73.8%.

### Correlations between DTI parameters and FAB

As the total FAB score was the only cognitive test able to differentiate patients with SIVD from those with AD, we performed partial correlation analysis, controlling for age and education, between the FA/MD values of all ROIs and the total FAB score in order to identify their associations (Table 5). Overall (SIVD + AD; *n* = 75), there were positive correlations between the total FAB score and FA values within the genu of the corpus callosum (*r* = 0.423; *p* < 0.001), bilateral forceps minor (right: *r* = 0.307; *p* = 0.013; left: *r* = 0.269; *p* = 0.030), left forceps major (*r* = 0.348; *p* = 0.005), left uncinate fasciculus (*r* = 0.258; *p* = 0.038), and right inferior longitudinal fasciculus (*r* = 0.350; *p* = 0.004). In comparison, inverse correlations were found between the total FAB score and MD values within the right superior longitudinal fasciculus (*r* = - 0.295; *p* = 0.017), genu (*r* = - 0.368; *p* = 0.003) and body (*r* = - 0.255; *p* = 0.040) of the corpus callosum, bilateral forceps minor (right: *r* = - 0.311; *p* = 0.012; left: *r* = - 0.353; *p* = 0.004), right uncinate fasciculus (*r* = - 0.263; *p* = 0.034), and right inferior longitudinal fasciculus (*r* = - 0.275; *p* = 0.027). Partial correlation analysis conducted among the patients with SIVD (*n* = 35) showed that total FAB score was correlated with FA values within the forceps minor (*r* = 0.455; *p* = 0.015), uncinate fasciculus (*r* = 0.435; *p* = 0.021), and inferior longitudinal fasciculus (*r* = 0.448; *p* = 0.017) of the right side. Among the patients with AD (*n* = 40), total FAB score was correlated with FA values within the genu of corpus callosum (*r* = 0.340; *p* = 0.045). In contrast, MD values of both dementia subgroups showed no significant correlations with FAB performances.

**Table 5:** Correlations between diffusion tensor imaging parameters and Frontal Assessment Battery.

	SIVD + AD (*n* = 75)		SIVD (*n* = 35)		AD (*n* = 40)	
Regions of Interest	FA	MD	FA	MD	FA	MD
Superior longitudinal fasciculus – Rt	0.197	- 0.295*	- 0.084	- 0.014	0.104	- 0.278
– Lt	0.099	- 0.046	- 0.084	0.251	0.019	0.044
Corpus callosum – Genu	0.423***	- 0.368**	0.343	- 0.281	0.340*	- 0.310
– Body 1	0.215	- 0.255*	0.267	- 0.137	0.038	- 0.189
– Body 2	0.009	- 0.055	- 0.022	0.065	- 0.153	- 0.014
– Body 3	0.115	- 0.191	0.174	- 0.185	- 0.041	- 0.104
– Splenium	0.215	- 0.199	0.189	- 0.177	0.082	- 0.052
Forceps minor – Rt	0.307*	- 0.311*	0.455*	- 0.073	0.026	- 0.277
– Lt	0.269*	- 0.353**	0.275	- 0.150	0.118	- 0.210
Forceps major – Rt	0.236	- 0.298	0.336	- 0.039	- 0.004	- 0.307
– Lt	0.348**	- 0.233	0.197	- 0.050	0.276	- 0.180
Anterior thalamic radiation – Rt	0.188	- 0.140	0.193	- 0.140	0.015	0.156
– Lt	0.228	- 0.186	0.127	- 0.056	0.101	0.020
Uncinate fasciculus – Rt	0.143	- 0.263*	0.435*	- 0.119	- 0.167	- 0.184
– Lt	0.258*	- 0.137	0.186	0.304	0.162	- 0.260
Inferior longitudinal fasciculus – Rt	0.350**	- 0.275*	0.448*	- 0.246	0.081	- 0.114
– Lt	0.149	- 0.244	0.099	- 0.142	- 0.115	- 0.083
Cingulum – Rt	0.106	- 0.094	0.060	0.044	- 0.126	- 0.017
– Lt	0.151	- 0.104	- 0.027	0.325	0.222	- 0.298

***:*p* <0.001;

**:*p* <0.01;

*:*p* <0.05.

Data presented as *correlation coefficient*. AD: Alzheimer’s disease; SIVD: Subcortical ischemic vascular disease; FA: fractional anisotropy; MD: mean diffusivity; Rt: Right; Lt: Left.

## Discussion

In this study, we compared cognitive assessment test results and DTI parameters between patients with SIVD and AD during the early stage of disease. Our results support the effectiveness of DTI in differentiating patients with SIVD from those with AD as well as correlating executive function. We observed a clearly different DTI profile between the SIVD and AD groups on the basis of comparable disease severity and general cognition. Compared to the cognitive assessments, it was easier to use changes in FA and MD to differentiate SIVD from AD, highlighting the different microstructural changes as the fundamental pathogenesis in these two diseases.

There was a different spatial pattern of a reduction in FA and increase in MD in the pairwise comparisons between the SIVD/AD and NC groups. While the patients with SIVD exhibited nearly global changes compared to group level data, significant changes in the patients with AD were confined to the FA values within the corpus callosum, bilateral forceps major, and superior longitudinal fasciculus, anterior thalamic radiation, uncinate fasciculus, and cingulum of the left side. These tracts appeared to be those involving the limbic system in addition to several neocortical fibers. Another study on DTI also reported that patients with SIVD were prone to show global changes in both FA and MD, whereas patients with AD often demonstrated modification in FA and MD along the limbic circuits [23]. Moreover, both reports showed that the magnitude and regions of reductions in FA among SIVD patients outnumbered the regions of MD elevation in patients with AD [23]. In contrast to gross regional assessments and/or averaging selected fibers from bi-hemispheres, asymmetric white matter involvement and changes in individual fibers were identified through our tract-based spatial statistics method. These results are consistent with a *priori* knowledge that both white and gray matter pathologies occur in the very early course of AD [38,39], while the main imaging features identified in patients with SIVD are lacunar infarcts and white matter T2-hyperintensities [12], to which DTI parameters are expected to be sensitive. As dementia represents a continuum of disease progression, our findings support that AD and SIVD have different etiopathogenic processes, and the potential of DTI to differentiate early pathological changes among patients with SIVD and AD and the normal ageing.

Two DTI parameters, FA and MD, were used in the current study. Although ROIs selections of DTI were devoid of regions being affected by white matter hyperintensities, their significant correlations and main effect of Fazekas scale as evident in MANCOVA indicated that white matter hyperintensities may have direct and/or indirect impact on DTI modifications. For example, FA and MD might alter along the regions under microischemia pathological process yet of normal appearances in conventional MRI. FA/MD values are also expected to change when there are lesions around the ROIs or in a distant location along the fibers. Theoretically, FA measures the overall directionality of water diffusion and reflects the complexity of cytoskeletal architecture, whereas MD represents a marker of neurodegeneration which reflects a decrease in membrane or other barriers to free water diffusion [39]. The exact underlying neurobiological properties behind these two DTI parameters remain unclear, as currently published results vary considerably according to the physiological/pathological condition and the stage of dementia. For example, one study focusing on brain maturation suggested that FA was sensitive to increases in axonal growth and myelination [40], while another DTI study including patients with multiple sclerosis reported that diffusion parameters were sensitive to destructive lesions, but that changes in anisotropy were most profound in inflammatory lesions [41]. In a cohort study of mild cognitive impairment where FA and three other additional diffusion parameters were investigated, FA was shown to be most sensitive to differentiate patterns of white matter changes as well as the subtype of mild cognitive impairment [42]. Consistent with these findings, another study focusing on DTI parameters among the normal aging and amnestic patients with mild cognitive impairment and AD also suggested that FA appeared to be more sensitive than MD and to have a better correlation with memory performance [43]. However, another cohort study reported opposite findings, in which MD indices were more sensitive than FA in detecting early pathological changes in patients with amnestic mild cognitive impairment [44]. Furthermore, another study including both patients with subjective and mild cognitive impairment indicated that although FA and MD could predict future cognitive decline, only MD could significantly predict transformation to dementia and medial temporal lobe atrophy [45]. Our results support that FA is a sensitive marker to early detect AD-related pathology, where a significant reduction in FA with unchanged MD occurred within the genu/splenium of the corpus callosum and several other associated fibers (i.e., bilateral forceps major and the left superior longitudinal fasciculus/anterior thalamic radiation/uncinate fasciculus/cingulum). As FA reflects directional dependence of selected fiber tracts, significant reductions in FA in the white matter without differences in MD may indicate changes in the axonal membrane as well as myelin integrity [46].

There was considerable overlap of the ROIs with reductions in FA and increases in MD in the patients with SIVD. The main pathogenesis is thought to be an ongoing neurodegenerative process, as both myelin and axonal damage occurs. Our results are consistent with previous studies, in that extensive arteriolosclerosis, lacunes/microinfarcts, and demyelinating changes have been reported to be the major pathological findings in patients with SIVD [47]. Deramecourt et al. proposed that vessel wall modifications such as arteriolosclerosis are the most common and earliest pathological changes in SIVD. These changes are then followed by perivascular spacing with lacunar and regional microinfarcts occurring as consequent but independent processes. Deep cerebral structures and white matter appear to be most vulnerable due to their end-artery properties, which are almost devoid of anastomoses [47]. These mechanisms subsequently cause both myelin and axonal damage, as reflected by changes in FA and MD. As such neuropathology often progresses with a slow course, DTI measurement can allow for the early detection of microstructural changes and the prompt reduction of vascular risk factors and cognitive decline.

In the comparisons between the NC and SIVD/AD groups, some regions still had increased MD with no changes in FA modifications (i.e., left forceps minor and right uncinate fasciculus in the patients with SIVD, and left superior longitudinal fasciculus, left forceps minor, right forceps major, and bilateral uncinate fasciculus in the patients with AD). This pattern of changes in MD and FA indicates widespread tissue damage resulting in a generalized increase in extracellular space due, for example, to the loss of axons and/or neurons as expected in Wallerian degeneration [48].

In particular, FA values within three selected regions in our DTI analysis showed acceptable clinical value in discriminating between patients with SIVD and those with AD (71.8% overall correct classification; sensitivity 69.4%; specificity 73.8%). However, differentiating these two subtypes of dementia using a single neuroimaging tool remains a challenge, as the discriminative power may be confounded by patient group selection as well as different imaging protocols. A previous volumetric brain imaging study showed that hippocampal volume alone could classify 63% of patients with SIVD from those with AD, but with only 50% sensitivity and 73% specificity [49]. The discriminative power could be improved (83% overall classification rate; sensitivity 72%; specificity 91%) by adding comprehensive values of brain segmentation analysis but not volumetric assessment of the entorhinal cortex. Although some studies have described spatial patterns of changes in DTI parameters in patients with SIVD and AD [23, 50], few studies have investigated the discriminative power of DTI parameters between patients with SIVD and those with AD.

We also provide evidence of the role of changes in white matter integrity within the bilateral forceps minor, genu and body of the corpus callosum, bilateral uncinate fasciculus, left forceps major, and right superior/inferior longitudinal fasciculus with regards to FAB performance. Of note, both FA and MD evaluations suggested that microstructural changes within the bilateral forceps minor and genu of the corpus callosum was responsible for FAB performance. The forceps minor represents interhemispheric fibers extending through the genu of the corpus callosum that govern projections toward the prefrontal lobes. They are considered to govern executive function as well as hemispheric specialization and interactions due to their widespread projections to prefrontal cortices [51]. However, the cognitive implementation of FAB is not limited to the frontal regions itself, and significant correlations with other major tracts (i.e., the uncinate fasciculus, forceps major, and superior/inferior longitudinal fasciculus) were identified in the current study. Such observation raises the possibility that disconnection between frontal and other regions may also contribute to the executive dysfunction in patients with SIVD.

There are several limitations to the current study. First, although we addressed the discernible DTI parameters and FAB performance between the patients with SIVD and those with AD during the early stage, we lacked data related to the pathophysiological biomarkers of AD (i.e., cerebrospinal fluid tau protein and Abeta42). Second, some of our ROIs were drawn in the crossing-fiber locations (e.g., forceps major) or regions adjacent to the gray matter (e.g., anterior thalamic radiation), potential partial-volume effect should be put into considerations on current DTI study interpretation. Third, as differentiating between a CDR score of 0.5 and 1 is sometimes regarded to be challenging with regard to the informants’ reports, additional longitudinal studies regarding patients with a CDR score of 0.5 should be conducted to confirm the clinical significance of discernible changes in DTI parameters/FAB performance during the early stages of AD and SIVD.

## Conclusions

Our findings suggest the effectiveness of DTI parameters in distinguishing patients with the early stage of AD and SIVD, as reflected by the discernible changes in spatial distribution and magnitude of significant modification in DTI parameters. Critical relation between white matter hyperintensities and DTI parameters was identified. The disconnection of white matter in the patients with SIVD appeared to be more profound than that in the patients with AD. The FA values within three ROIs (the left forceps major, left anterior thalamic radiation, and the genu of the corpus callosum) provided a satisfactory discriminative power (71.8% overall correct classification; sensitivity 69.4%; specificity 73.8%). The FAB may serve as an additional cognitive marker to differentiate patients with SIVD from those with AD. That the white matter integrity within the bilateral forceps minor, genu and body of the corpus callosum, bilateral uncinate fasciculus, left forceps major, and right superior/inferior longitudinal fasciculus was associated with FAB performance highlights the fundamental implication of executive function and relevant circuit connectivity.

## Supporting information

S1 Appendix Correlation between Fazekas scale and diffusion tensor imaging parameters. ***:*p* <0.001; **:*p* <0.01; *:*p* <0.05. Data presented as Spearman’s rank correlation coefficient. AD: Alzheimer’s disease; SIVD: Subcortical ischemic vascular disease; FA: fractional anisotropy; MD: mean diffusivity; Rt: Right; Lt: Left.
(DOC)

## References

[1] World Health Organization. Dementia Fact sheet. 2016. http://www.who.int/mediacentre/factsheets/fs362/en/

[2] Herrera E Jr, Caramelli P, Silveira AS, Nitrini R. Epidemiologic survey of dementia in a community-dwelling Brazilian population. Alzheimer Dis Assoc Disord. 2002;16(2):103–8. 12040305 10.1097/00002093-200204000-00007

[3] Englund E, Brun A. Histopathology. White matter changes in dementia of Alzheimer's type: the difference in vulnerability between cell compartments. 1990; 16(5):433–9. 10.1111/j.1365-2559.1990.tb01542.x 2361659

[4] O’Brien JT, Ames D, Schwietzer I. White matter in depression and Alzheimer’s disease: a review of magnetic resonance imaging studies. International Journal of Geriatric Psychiatry. 1996;11:681–94.

[5] Kalaria RN, Ballard C. Overlap between pathology of Alzheimer disease and vascular dementia. Alzheimer Dis Assoc Disord. 1999;13 Suppl 3:S115–23. 10609690 10.1097/00002093-199912003-00017

[6] Érica MLP. Role of neuropsychological assessment in the differential diagnosis of Alzheimer’s disease and vascular dementia. Dementia & Neuropsychologia 2009;3(3):214–221, 29213631 10.1590/S1980-57642009DN30300007 PMC5618976

[7] Golden Z, Bouvier M, Selden J, Mattis K, Todd M, Golden C. Differential performance of Alzheimer's and vascular dementia patients on a brief battery of neuropsychological tests. Int J Neurosci. 2005; 115(11):1569–77. 10.1080/00207450590957953 16223702

[8] Yuspeh RL, Vanderploeg RD, Crowell TA, Mullan M. Differences in executive functioning between Alzheimer's disease and subcortical ischemic vascular dementia. J Clin Exp Neuropsychol. 2002;24(6):745–54. 10.1076/jcen.24.6.745.8399 12424649

[9] Boutoleau-Bretonnière C, Lebouvier T, Delaroche O, Lamy E, Evrard C, Charriau T, et al Value of neuropsychological testing, imaging, and CSF biomarkers for the differential diagnosis and prognosis of clinically ambiguous dementia. J Alzheimers Dis. 2012;28(2):323–36. 10.3233/JAD-2011-110761 22008265

[10] Matioli MN, Caramelli P. Limitations in differentiating vascular dementia from Alzheimer's disease with brief cognitive tests. Arq Neuropsiquiatr. 2010;68(2):185–8. 20464282 10.1590/s0004-282x2010000200006

[11] Jellinger KA. The pathology of "vascular dementia": a critical update. J Alzheimers Dis. 2008;14(1):107–23. 18525132 10.3233/jad-2008-14110

[12] Erkinjuntti T, Inzitari D, Pantoni L, Wallin A, Scheltens P, Rockwood K, et al Research criteria for subcortical vascular dementia in clinical trials. J Neural Transm Suppl. 2000;59:23–30. 10961414 10.1007/978-3-7091-6781-6_4

[13] Altamura C, Scrascia F, Quattrocchi CC, Errante Y, Gangemi E, Curcio G Clin J, et al Regional MRI Diffusion, White-Matter Hyperintensities, and Cognitive Function in Alzheimer's Disease and Vascular Dementia. Neurol. 2016;12(2):201–8. 10.3988/jcn.2016.12.2.201 PMC4828567 27074295

[14] Tu MC, Huang CW, Chen NC, Chang WN, Lui CC, Chen CF, et al Hyperhomocysteinemia in Alzheimer dementia patients and cognitive decline after 6 months follow-up period. Acta Neurol Taiwan. 2010;19(3):168–77. 20824536

[15] Ylikoski A, Erkinjuntti T, Raininko R, Sarna S, Sulkava R, Tilvis R. White matter hyperintensities on MRI in the neurologically nondiseased elderly. Analysis of cohorts of consecutive subjects aged 55 to 85 years living at home. Stroke. 1995;26(7):1171–7. 7604409 10.1161/01.str.26.7.1171

[16] Gunning-Dixon FM, Raz N. The cognitive correlates of white matter abnormalities in normal aging: a quantitative review. Neuropsychology. 2000;14(2):224–32. 10791862 10.1037//0894-4105.14.2.224

[17] Alexander AL, Lee JE, Lazar M, Field AS. Diffusion tensor imaging of the brain. Neurotherapeutics. 2007 7;4(3):316–29. 10.1016/j.nurt.2007.05.011 17599699 PMC2041910

[18] Naggara O, Oppenheim C, Rieu D, Raoux N, Rodrigo S, Dalla Barba G, et al Diffusion tensor imaging in early Alzheimer's disease. Psychiatry Res. 2006;146(3):243–9. 10.1016/j.pscychresns.2006.01.005 16520023

[19] Mielke MM, Kozauer NA, Chan KC, George M, Toroney J, Zerrate M, et al Regionally-specific diffusion tensor imaging in mild cognitive impairment and Alzheimer's disease. Neuroimage. 2009;46(1):47–55. 10.1016/j.neuroimage.2009.01.054 19457371 PMC2688089

[20] Cherubini A, Péran P, Spoletini I, Di Paola M, Di Iulio F, Hagberg GE, et al Combined volumetry and DTI in subcortical structures of mild cognitive impairment and Alzheimer's disease patients. J Alzheimers Dis. 2010;19(4):1273–82. 10.3233/JAD-2010-091186 20308792

[21] Mayzel-Oreg O, Assaf Y, Gigi A, Ben-Bashat D, Verchovsky R, Mordohovitch M, et al High b-value diffusion imaging of dementia: application to vascular dementia and alzheimer disease. J Neurol Sci. 2007;257(1–2):105–13. 10.1016/j.jns.2007.01.048 17360001

[22] Sugihara S, Kinoshita T, Matsusue E, Fujii S, Ogawa T. Usefulness of diffusion tensor imaging of white matter in Alzheimer disease and vascular dementia. Acta Radiol. 2004;45(6):658–63. 15587425 10.1080/02841850410008388

[23] Fu JL, Zhang T, Chang C, Zhang YZ, Li WB. The value of diffusion tensor imaging in the differential diagnosis of subcortical ischemic vascular dementia and Alzheimer's disease in patients with only mild white matter alterations on T2-weighted images. Acta Radiol. 2012;53(3):312–7. 10.1258/ar.2011.110272 22416261

[24] Morris JC. The Clinical Dementia Rating (CDR): current version and scoring rules. Neurology. 1993;43(11):2412–4. 10.1212/wnl.43.11.2412-a 8232972

[25] Shyu YI, Yip PK. Factor structure and explanatory variables of the Mini-Mental State Examination (MMSE) for elderly persons in Taiwan. J Formos Med Assoc. 2001;100(10):676–83. 11760373

[26] Hachinski VC, Iliff LD, Zilhka E, Du Boulay GH, McAllister VL, Marshall J, et al Cerebral blood flow in dementia. Arch Neurol. 1975;32(9):632–7. 1164215 10.1001/archneur.1975.00490510088009

[27] Albert MS, DeKosky ST, Dickson D, Dubois B, Feldman HH, Fox NC, et al The diagnosis of mild cognitive impairment due to Alzheimer's disease: recommendations from the National Institute on Aging-Alzheimer's Association workgroups on diagnostic guidelines for Alzheimer's disease. Alzheimers Dement. 2011;7(3):270–9. 10.1016/j.jalz.2011.03.008 21514249 PMC3312027

[28] Chobanian AV, Bakris GL, Black HR, Cushman WC, Green LA, Izzo JL Jr, et al The Seventh Report of the Joint National Committee on Prevention, Detection, Evaluation, and Treatment of High Blood Pressure: the JNC 7 report. JAMA. 2003;289(19):2560–72. 10.1001/jama.289.19.2560 12748199

[29] American Diabetes Association AD. Standards of medical care indiabetes—2015. Diabetes Care 2015;38:S1–S94.

[30] Levey AS, Bosch JP, Lewis JB, Greene T, Rogers N, Roth D. A more accurate method to estimate glomerular filtration rate from serum creatinine: a new prediction equation. Modification of Diet in Renal Disease Study Group. Ann Intern Med. 1999;130(6):461–70. 10075613 10.7326/0003-4819-130-6-199903160-00002

[31] Snyder S, Pendergraph B. Detection and evaluation of chronic kidney disease. Am Fam Physician. 2005 11 1;72(9):1723–32. 16300034

[32] Cannon CP, Brindis RG, Chaitman BR, Cohen DJ, Cross JT Jr, Drozda JP Jr, et al 2013 ACCF/AHA key data elements and definitions for measuring the clinical management and outcomes of patients with acute coronary syndromes and coronary artery disease: a report of the American College of Cardiology Foundation/American Heart Association Task Force on Clinical Data Standards (Writing Committee to Develop Acute Coronary Syndromes and Coronary Artery Disease Clinical Data Standards). Circulation. 2013;127(9):1052–89. 10.1161/CIR.0b013e3182831a11 23357718

[33] Chang CC, Kramer JH, Lin KN, Chang WN, Wang YL, Huang CW, et al Validating the Chinese version of the Verbal Learning Test for screening Alzheimer's disease.J Int Neuropsychol Soc. 2010;16(2):244–51. 10.1017/S1355617709991184 20003579 PMC3767760

[34] Wechsler D. Wechsler Memory Scale. 3^rd^ ed San Antonio, TX: Psychological Corportation;1997

[35] Dubois B, Slachevsky A, Litvan I, Pillon B. The FAB: a Frontal Assessment Battery at bedside. Neurology. 2000;55(11):1621–6. 11113214 10.1212/wnl.55.11.1621

[36] Shulman KI. Clock-drawing: is it the ideal cognitive screening test? Int J Geriatr Psychiatry. 2000;15(6):548–61. 10861923 10.1002/1099-1166(200006)15:6<548::aid-gps242>3.0.co;2-u

[37] Fazekas F, Chawluk JB, Alavi A, Hurtig HI, Zimmerman RA. MR signal abnormalities at 1.5 T in Alzheimer's dementia and normal aging. AJR Am J Roentgenol. 1987;149(2):351–6. 10.2214/ajr.149.2.351 3496763

[38] Montine TJ, Phelps CH, Beach TG, Bigio EH, Cairns NJ, Dickson DW, et al National Institute on Aging-Alzheimer's Association guidelines for the neuropathologic assessment of Alzheimer's disease: a practical approach. Acta Neuropathol. 2012;123(1):1–11. Epub 2011 Nov 20. 10.1007/s00401-011-0910-3 22101365 PMC3268003

[39] Alves GS, Oertel Knöchel V, Knöchel C, Carvalho AF, Pantel J, Engelhardt E, et al Integrating retrogenesis theory to Alzheimer's disease pathology: insight from DTI-TBSS investigation of the white matter microstructural integrity. Biomed Res Int. 2015;2015:291658 10.1155/2015/291658 25685779 PMC4320890

[40] Lebel C, Walker L, Leemans A, Phillips L, Beaulieu C. Microstructural maturation of the human brain from childhood to adulthood. Neuroimage. 2008;40(3):1044–55. 10.1016/j.neuroimage.2007.12.053 18295509

[41] Werring DJ, Clark CA, Barker GJ, Thompson AJ, Miller DH. Diffusion tensor imaging of lesions and normal-appearing white matter in multiple sclerosis. Neurology. 1999;52(8):1626–32. 10331689 10.1212/wnl.52.8.1626

[42] Haller S, Missonnier P, Herrmann FR, Rodriguez C, Deiber MP, Nguyen D, et al Individual classification of mild cognitive impairment subtypes by support vector machine analysis of white matter DTI. AJNR Am J Neuroradiol. 2013;34(2):283–91. 10.3174/ajnr.A3223 22976235 PMC7965106

[43] Bosch B, Arenaza-Urquijo EM, Rami L, Sala-Llonch R, Junqué C, Solé-Padullés C, et al Multiple DTI index analysis in normal aging, amnestic MCI and AD. Relationship with neuropsychological performance. Neurobiol Aging. 2012;33(1):61–74. 10.1016/j.neurobiolaging.2010.02.004 20371138

[44] O'Dwyer L, Lamberton F, Bokde AL, Ewers M, Faluyi YO, Tanner C, et al Multiple indices of diffusion identifies white matter damage in mild cognitive impairment and Alzheimer's disease. PLoS One. 2011;6(6):e21745 10.1371/journal.pone.0021745 21738785 PMC3128090

[45] Selnes P, Aarsland D, Bjørnerud A, Gjerstad L, Wallin A, Hessen E, et al Diffusion tensor imaging surpasses cerebrospinal fluid as predictor of cognitive decline and medial temporal lobe atrophy in subjective cognitive impairment and mild cognitive impairment. J Alzheimers Dis. 2013;33(3):723–36. 10.3233/JAD-2012-121603 23186987

[46] Beaulieu C. The basis of anisotropic water diffusion in the nervous system—a technical review. NMR Biomed. 2002;15(7–8):435–55. 10.1002/nbm.782 12489094

[47] Kalaria RN. Neuropathological diagnosis of vascular cognitive impairment and vascular dementia with implications for Alzheimer's disease. Acta Neuropathol. 2016;131(5):659–85. 10.1007/s00401-016-1571-z 27062261 PMC4835512

[48] Di Paola M, Di Iulio F, Cherubini A, Blundo C, Casini AR, Sancesario G, et al When, where, and how the corpus callosum changes in MCI and AD: a multimodal MRI study. Neurology. 2010;74(14):1136–42. 10.1212/WNL.0b013e3181d7d8cb 20368633

[49] Du AT, Schuff N, Laakso MP, Zhu XP, Jagust WJ, Yaffe K, et al Effects of subcortical ischemic vascular dementia and AD on entorhinal cortex and hippocampus. Neurology. 2002;58(11):1635–41. 12058091 10.1212/wnl.58.11.1635 PMC1820858

[50] Chen TF, Lin CC, Chen YF, Liu HM, Hua MS, Huang YC, et al Diffusion tensor changes in patients with amnesic mild cognitive impairment and various dementias. Psychiatry Res. 2009;173(1):15–21. 10.1016/j.pscychresns.2008.09.002 19442496

[51] Hofer S, Frahm J. Topography of the human corpus callosum revisited--comprehensive fiber tractography using diffusion tensor magnetic resonance imaging. Neuroimage. 2006;32(3):989–94. 10.1016/j.neuroimage.2006.05.044 16854598

