# Supplementary material for: Effectiveness of diffusion tensor imaging in differentiating early-stage subcortical ischemic vascular disease, Alzheimer’s disease and normal ageing
Source: PLoS One. 2017 Apr 7;12(4):e0175143. doi: 10.1371/journal.pone.0175143 (PMC5384760; doi:10.1371/journal.pone.0175143)
Supplement: S1 Appendix — ***:p <0.001; **:p <0.01; *:p <0.05. Data presented as Spearman’s rank correlation coefficient. AD: Alzheimer’s disease; SIVD: Subcortical ischemic vascular disease; FA: fractional anisotropy; MD: mean diffusivity; Rt: Right; Lt: Left. (DOC) [file pone.0175143.s001.doc]

**Appendix. Correlation between Fazekas** scale and diffusion tensor imaging parameters.

|  | Total score | | Periventricular  white matter hyperintensities | | Deep  white matter hyperintensities | |
| --- | --- | --- | --- | --- | --- | --- |
| Regions of Interest | FA | MD | FA | MD | FA | MD |
| Superior longitudinal fasciculus – Rt | - 0.431*** | 0.685*** | - 0.316** | 0.691*** | - 0.445*** | 0.542*** |
| – Lt | - 0.329** | 0.422*** | - 0.206 | 0.373** | - 0.371** | 0.379** |
| Corpus callosum – Genu | - 0.330** | 0.281* | - 0.401*** | 0.359** | - 0.201 | 0.156 |
| – Body 1 | - 0.376** | 0.394*** | - 0.353** | 0.342** | - 0.320** | 0.360** |
| – Body 2 | - 0.303* | 0.270* | - 0.324** | 0.278* | - 0.223 | 0.208 |
| – Body 3 | - 0.346** | 0.321** | - 0.322** | 0.281* | - 0.297* | 0.292* |
| – Splenium | - 0.221 | 0.261* | - 0.207 | 0.233 | - 0.188 | 0.232 |
| Forceps minor – Rt | - 0.504*** | 0.510*** | - 0.426*** | 0.417*** | - 0.472*** | 0.490*** |
| –Lt | - 0.331** | 0.652*** | - 0.266* | 0.557*** | - 0.322** | 0.604*** |
| Forceps major – Rt | - 0.281* | 0.511*** | - 0.351** | 0.427*** | - 0.163 | 0.481*** |
| – Lt | - 0.394*** | 0.522*** | - 0.388*** | 0.454*** | - 0.320** | 0.476*** |
| Anterior thalamic radiation – Rt | - 0.333** | 0.481*** | - 0.293* | 0.482*** | - 0.302* | 0.382** |
| – Lt | - 0.386*** | 0.438*** | - 0.320** | 0.384** | - 0.366** | 0.397*** |
| Uncinate fasciculus – Rt | - 0.103 | 0.416*** | - 0.072 | 0.403*** | - 0.110 | 0.344** |
| – Lt | - 0.167 | 0.343** | - 0.164 | 0.370** | - 0.137 | 0.250* |
| Inferior longitudinal fasciculus – Rt | - 0.361** | 0.336** | - 0.447*** | 0.349** | - 0.211 | 0.257* |
| – Lt | - 0.445*** | 0.433*** | - 0.425*** | 0.384** | - 0.373** | 0.388*** |
| Cingulum – Rt | - 0.280* | 0.285* | - 0.293* | 0.280* | - 0.212 | 0.232 |
| – Lt | 0.007 | 0.177 | - 0.047 | 0.027 | 0.054 | 0.275* |

***:*p* <0.001; **:*p* <0.01; *:*p* <0.05. Data presented as Spearman’s rank correlation coefficient. AD: Alzheimer’s disease; SIVD: Subcortical ischemic vascular disease; FA: fractional anisotropy; MD: mean diffusivity; Rt: Right; Lt: Left.
